# New insights into the mechanical properties of *Acanthamoeba castellanii *cysts as revealed by phonon microscopy

**DOI:** 10.1364/BOE.10.002399

**Published:** 2019-04-15

**Authors:** Fernando Pérez-Cota, Richard J. Smith, Hany M. Elsheikha, Matt Clark

**Affiliations:** 1 Optics and Photonics Group, Faculty of Engineering, University of Nottingham, University Park, Nottingham, NG7 2RD, United Kingdom; 2 Faculty of Medicine and Health Sciences, School of Veterinary Medicine and Science, University of Nottingham, Sutton Bonington Campus, Loughborough, LE12 5RD, United Kingdom

## Abstract

The single cell eukaryotic protozoan *Acanthamoeba castellanii* exhibits a remarkable ability to switch from a vegetative trophozoite stage to a cystic form, in response to stressors. This phenotypic switch involves changes in gene expression and synthesis of the cell wall, which affects the ability of the organism to resist biocides and chemotherapeutic medicines. Given that encystation is a fundamental survival mechanism in the life cycle of *A. castellanii*, understanding of this process should have significant environmental and medical implications. In the present study, we investigated the mechanism of *A. castellanii* encystation using a novel phonon microscopy technique at the single cell level. Phonon microscopy is an emerging technique to image cells using laser-generated sub-optical wavelength phonons. This imaging modality can image with contrast underpinned by mechanical properties of cells at an optical or higher resolution. Our results show that the Brillouin frequency, a shift of the colour of light induced by phonons, evolves in three well defined frequency bands instead of a simple shift in frequency. These observations confirm previous results from literature and provide new insights into the capacity of *A. castellanii* cyst to react quickly in harsh environments.

## 1. Introduction

*Acanthamoeba castellanii* is a free-living protozoan with two life cycle stages: an active trophozoite and a dormant cyst. *A. castellanii* is a complex organism that exhibits tolerance to adverse environmental conditions by undergoing changes in morphology from a trophozoite to a cyst. In this cystic form, *A. castellanii* can survive extreme conditions such as starvation, temperature, high osmolarity and desiccation [1]. The ability of the cystic form to excyst back to the trophozoite (active) state in favourable conditions, shows the remarkable ability of *A. castellanii* to survive harsh conditions.

This organism can cause Granulomatous Amoebic Encephalitis (GAE), a rare but often fatal disease if not timely treated [2], and *A. castellanii* Keratitis (AK) a painful swelling of the cornea leading to damage and even blindness. There is an increasing concern about GAE, due to the rise in the prevalence of *A. castellanii* infection and the growing number of immunocompromised individuals [3]. AK is mostly related to contact lens use, with over 90% of the cases having this as a risk factor [4], but can also occur in non-contact lens wearers.

The cyst wall of *A. castellanii* protects this organism against harmful conditions, as well as against most pharmacological treatment, which makes infection difficult to treat [5]. Current drugs have limited efficacy against the cystic stage and pose a risk to infected individuals due to their side-effects. Therefore, understanding the encystation process is important to develop new drugs to tackle these limitations. Encystation of *A. castellanii*, the underlying molecular mechanisms of this phenomenon, the relationship between gene expression and chemical composition of the cyst wall are rapidly expanding areas of research [6–11]. The cyst wall of *A. castellanii* undergoes remarkable changes in its mechanical properties, defined by its biochemical composition, during encystation [12]. However, knowledge regarding the mechanical properties of *A. castellanii* cysts has been limited, due to the inability to examine phenotypic transformation in situ with resolution high enough for single cell observations.

Imaging and probing mechanical properties of cells with phonons offers intriguing possibilities for understanding fundamental mechanisms of cell biology. Among many features, its contrast is governed by the mechanical properties of the specimen and its lateral resolution is governed by the optical system used for imaging [13–15]. However, imaging has proved challenging particularly when dealing with cells in liquid and with relatively high numerical apertures. Phonon microscopy, a novel approach for imaging using phonons [16, 17], offers solutions to some of the limitations of the phonon technologies. The phonon microscope is enabled by a novel opto-acoustic transducer that is engineered to reduce laser damage while increasing signal to noise ratio of the detected Brillouin scattering signal. This enables high resolution imaging with faster acquisition times and provides a mean to elucidate the fundamental changes that occur in *A. castellanii* during encystation.

In this study, we investigated the process of *A. castellanii* encystation, for the first time, using Brillouin scattering and phonon microscopy. We observed significant changes in the Brillouin frequency at specific time points during encystation. These changes were consistent with the previously reported features associated with encystation of this organism [10, 12, 18]. Our data provide the first proof-of-concept that purely biophysical tool, such as phonon microscopy can be harnessed to uncover label-free biomarkers associating the phenotype of the cyst with its chemical properties at a single organism level.

## 2. Methods

### 2.1. Sample preparation

*Acanthamoeba castellanii* T4 genotype (American Type Culture Collection; ATCC 30011) was grown in 20ml of peptone glucose yeast (PYG) medium [proteose-peptone 0.75% (w/v), yeast extract 0.75% (w/v) and glucose 1.5% (w/v)] in T-75 tissue culture flasks at 25°C in a humidified Stuart hybridization/shaker table top oven without rocking [19]. The culture media was refreshed 15-20 hours before each experiment to ensure that up to 95% of the parasites were vegetative trophozoites [20].

To prepare *A. castellanii* cysts, encystation was induced by suspending 5x10^6^ *A. castellanii* trophozoites in 15 ml encystation buffer, which consists of phosphate buffered saline (PBS) containing 50 mM MgCl2 and 10% glucose, per T-75 tissue culture flask and incubating at 25°C for 4 days. Before induction of encystation and at 1, 3, 5, 24, and 48 hours after encystation, 1 ml of the encystation buffer containing the encysting *A. castellanii* was collected and centrifuged at 3000xg for 5 min. After discarding the supernatant, the pellet containing the cysts was washed twice in PBS by centrifugation at the same centrifugational conditions. Then, *A. castellanii* cysts were suspended in ∼200 μ l of water and spread on the surface of coverslips and left to air-dry for about 20 min. The dried film of *A. castellanii* were fixed in 4% paraformaldehyde (PFA) for 30 minutes, followed by PBS washing thrice (3 ml each) to remove the traces of the PFA. The PFA-fixed cysts were adherent to the surface of the opto-acoustic transducer (OAT), however cells were rehydrated afterwards for imaging. Each substrate was then mounted in a two coverslip chamber (chamlide-tc) filled with buffer medium for imaging using the phonon microscope.

In a parallel experiment, air-dried smear of *A. castellanii* cysts, at the above time points post encystation were subjected to staining with Calcuflour White (CW) dye (Sigma) which was freshly prepared in distilled water to a concentration of 25μg ml^−1^. Cysts were stained with CW for 5 min at ambient temperature, followed by washing the stained cysts on the surface of the coverslip with distilled water. Stained cysts were examined under Leica microscope (Leica Microsystems, Wetzlar, Germany). Cells were imaged retaining the same acquisition parameters across all time points.

### 2.2. Phonon microscope

**Fig. 1:**
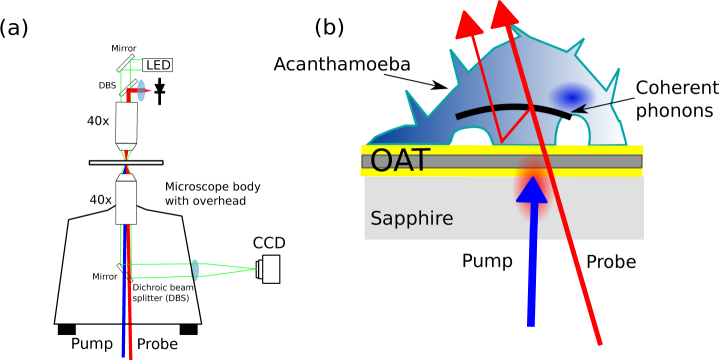
Phonon microscope. a) Experimental setup. Menlo C-fibre ASOPS laser beams are directed to the sample using a microscope body. The transmitted light is collected using an overhead optical assembly and captured by an oscilloscope. b) The opto-acoustic transducer (OAT) is made of gold and indium tin oxide layers and is optimised for absorption of pump light and transmission of probe light. The generated phonon field interacts with the probe light producing a TRBS signal.

Phonons are generated by the absorption of short light pulses in metallic thin films [21] and detected by interferometry in a technique commonly referred as time-resolved Brillouin scattering (TRBS) [22]. The phonon microscope optimizes this process for single cell imaging [17, 23]. In this scheme the detection is performed in transmission and the thin-film is replaced with a multi-layer structured opto acoustic transducer (OAT) which protects the cell from pump light exposure and resonates both acoustically and optically to improve signal to noise ratio (see Fig. 1). A sapphire coverslip is used to reduce heating due to absorption of pump light.

The phonon microscope is built around an asynchronous optical sampling pump-probe system (ASOPS, see Fig. 1) [24]. This system controls two 150fs pulsed lasers (780nm for probe and 390nm for pump) with repetition rates of ∼100MHz and allows a delay between the lasers to be set and swept electronically without the need for a mechanical delay line. In our system the delay repetition rate is 10kHz so a measurement is taken every 100μs with a time resolution of 1ps. However the acquisition card samples the equivalent of 2ps for speed. The system uses typical average powers at the of 0.3mW at cell for the probe and 0.5 mW at the transducer for the pump. Ten thousand averages are taken per pixel at a speed of ∼3 points per second and a complete image was acquired in approximately two hours depending on number of pixels.

Compared to Brillouin microscopy [25], phonon microscopy uses the same fundamental mechanism to obtain a measure of the sound velocity: the frequency shift of photons that are scattered by phonons (Brillouin frequency). However signal generation in Brillouin microscopy is an inefficient process (one in every ∼10^10^-10^12^ photons contributes to the signal [26]) since it relies on thermally generated phonons within the sample and thus requires high optical fluxes to obtain sufficient SNR. Furthermore the method is intrinsically optically-limited in resolution because the thermal phonons are incoherent. In phonon microscopy the generation of coherent phonons (same wavelegnth and direction of propagation) along with an acoustically resonant cavity allows an increase of the signal with reduced exposure to optical power. This makes phonon microscopy ideal for single cell applications and even compatible with living cells at high resolutions. Since the time of flight is accessible through pump-probe configuration, it is possible to section axially with resolution given by the acoustic wavelength ($∼\lambda_{probe}$/2) independently of the numerical aperture of the system [17] and without confocal configurations.

### 2.3. Data processing

Data processing of the TBRS signals and scanning methods can be found in previous works [16, 17] but can be simplified as follows: The time vector was calculated by the ratio between repetition and delay rates and the raw data was cropped with respect to the temporal position of the coincidence peak (time at which pump and probe arrive simultaneously to the sample). The data were then fitted to a low order polynomial to remove the thermal relaxation. The signal was low pass filtered and its Fourier transform calculated to obtain its frequency. Repeating this process for each measurement produced a Brillouin frequency map.

The frequency maps obtained from the *A. castellanii* were processed to obtain their spectral content and observe its time evolution. Three measurements were performed for each time point each one from a different cyst. From the resultant images, the signal from the medium (5.1GHz) was filtered spatially by applying a mask. The remaining points where converted into a histogram where the amplitude of each bin were normalised to the number of samples in the histogram. The centre and amplitude of the distributions observed in the histograms were estimated by performing a fit of a sum of Gaussian curves (one for each observed distribution). Three distributions were found at approximately 5.4, 5.8 and 6.2GHz and with a standard deviation of ∼150MHz.

Weighting the intensity of each pixel in the frequency maps according to the fitted distributions, it was possible to create a RGB composite image for each time point where each distribution had its own colour: red for 5.4GHz, green for 5.8GHz and blue for 6.2GHz. The RGB composites show the spatial location of each frequency distribution.

The amplitudes of each distribution at each time point were fitted calculating their confidence intervals. The first two distributions were fitted to a decaying exponential while the 6.2GHz to a logistic function. The error on the determination of the amplitude of each distribution was estimated by simulating TBRS signals as decaying sine functions and was found to be significantly smaller (<5%) than the variations with time, hence error bars were not plotted.

## 3. Results and discussion

**Fig. 2:**
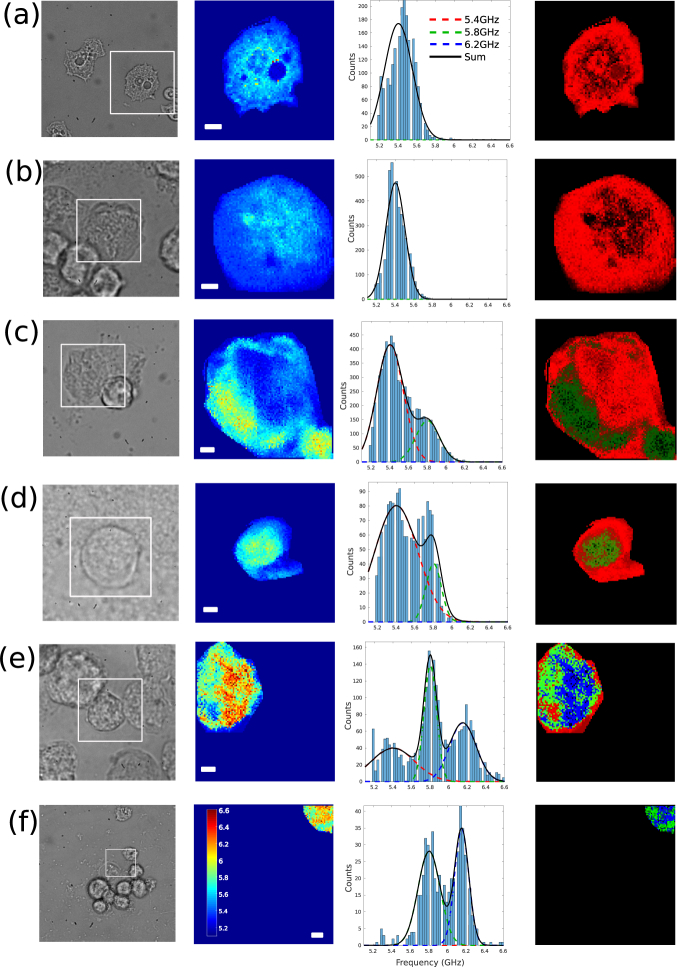
Brillouin frequency maps of *Acanthamoeba castellanii* at different time points. Images are presented in groups of four with optical images in gray scale, frequency images (scale bars: 5μm, same scale for all) in colour, histograms of the measured frequencies and composites. The histograms come only from the area of interest to remove contributions from the medium. Image groups a)-f) represent hours 0, 1,3, 5, 24 and 48 respectively. As time advances, the Brillouin frequency measured from the encysting amoebas increases. For the histograms, more than one frequency distribution appears and at 24Hr three distributions are clearly observable. Red, blue and green lines represent fits of the data to normal distributions. Composites were produced by identifying the distribution of origin for each pixel to assigning them a different colour. Composites represent the spatial location of each frequency distribution with red, green and blue corresponding to 5.4, 5.8 and 6.2 GHz respectively.

Figure 2 shows the phonon microscope images obtained from the time-series which are presented in groups of four. From left to right: optical brightfield image (left), Brillouin frequency (*f_B_*), frequency spectrum, and composites. The Brillouiin frequency *f_B_* is directly proportional to the sound velocity and a good marker for biology [27–29]. All images were acquired from different cells. As the encystation process advances, there was a clear broadening in the range of *f_B_* components. This change in Brillouin frequency is expected as a result of the encystation: the amoeba is losing volume, dehydrating and synthesising cellulose which causes compaction and stiffening.

**Fig. 3:**
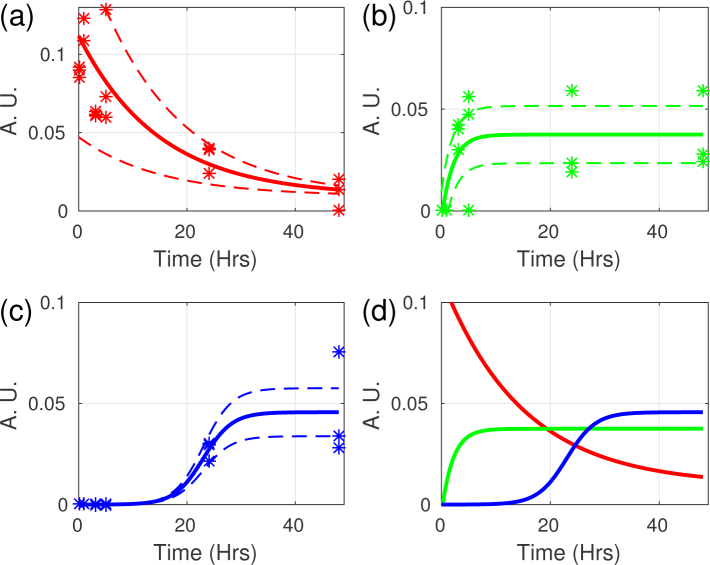
Progression of the amplitude Brillouin frequency distributions against time. The three different distributions have progressing amplitudes against time in different manner. The 5.4, 5.8 and 6.2GHz distributions are shown in (a),(b) and (c) respectively. The stars represent experimental data from individual organisms while the solid lines represent a fit of the data to an exponential decay (5.4 and 5.8GHz) or logistic function (6.2GHz). The dotted lines represent the confidence intervals of the fits. (d) Shows the combination of all distribution revealing complex dynamics.

Additional information can be obtained by observing the spectrum of Brillouin frequencies. By doing so, three clear distributions are observed across the time-series (see Fig. 2). To investigate the nature of these three frequency distributions, their amplitudes were plotted against time (see Fig. 3). The first distribution is centered around 5.4GHz (red dotted line) and with a standard deviation of a few hundred MHz. This distribution (see Fig. 3a) reduces in amplitude with time and almost disappears completely after ∼48Hrs. This coincides, in time-scales and microscopic observations, with the previously reported sub-encystation stage of degradation of the cytoplasmic elements [18]. During this stage, the *A. castellanii* experiences a reduction of volume: many cellular components are degraded providing raw materials for the formation of the cyst. Encysting *A. castellanii* also removes water and substantial quantities of material from their cytoplasm which is manifested as discharge of cellular debris. This suggests that the relative amplitude of this distribution (5.4GHz) is an indication of the presence of water as the Brillouin frequency is sensitive to hydration [30] and previously observed cells (which are rich in water) have their Brillouin frequencies in this range.

**Fig. 4:**
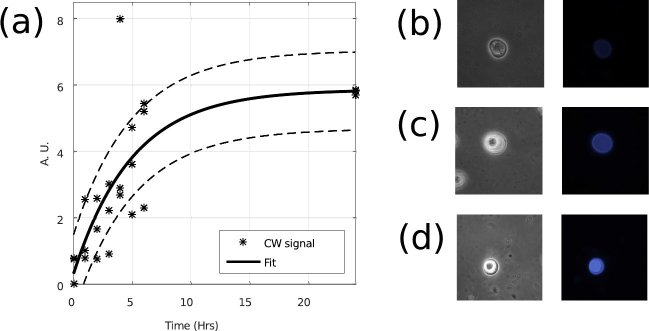
Progression of the amplitude of the cellulose signal against time during encystation. The trace in (a) shows the measured intensity of the cellulose signal. Here the stars represent experimental measurement of the fluorescence intensity while the solid lines represent a fit of the data to an exponential curve. The dotted lines represent the confidence intervals of the fit. Cellulose is observed to increase exponentially. Examples of different time points are presented in pairs with phase contrast images in gray scale and fluorescence images in colour. (a-d) Represent hours 3, 5 and 24 respectively.

The next stage in encystation is the generation of a cyst wall which is divided in two parts: the endocyst wall and exocyst wall which correlates with the synthesis of cellulose and an unidentified acid-resistant protein. Since cellulose is a well known component, it can be labelled by the use of fluorescent dyes. To investigate whether a cellulose signal correlates with any of the two later distributions presented in Fig. 3, an optical imaging experiment was performed in parallel to the phonon microscope imaging (see Fig. 2). In this experiment, CW dye was used to label the cellulose in the wall of *A. castellanii* (see methods). The result of cellulose staining indicates that the intensity of CW fluorescence increases significantly 2 hours after induction of encystation, reaching saturation at approximately 8 hours (see Fig. 4).

**Fig. 5:**
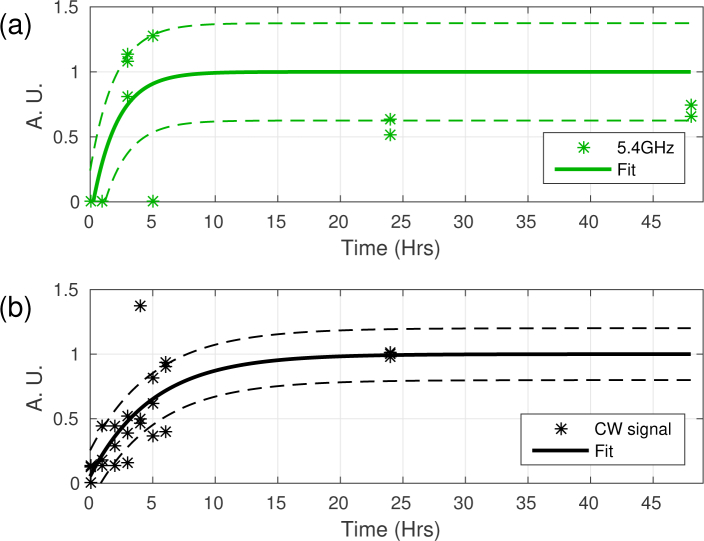
Comparison between the 5.8GHz distribution and CW signal.(a) 5.8GHz signal as seen in Fig. 3(b). (b) CW signal as seen in Fig. 4(a). Both signals increase between 3-10Hrs. This suggests that the 5.8GHz distribution arises from the synthesis of cellulose.

Figure 5 compares the results presented in Figs. 3(b) and 4. This shows that the 5.8GHz distribution obtained with phonon microscopy (green) correlates well with the cellulose signal obtained by fluorescence (black). This correlation suggests that the cellulose must have been synthesised quickly providing a significant physical barrier and this happens while degradation must have been still taking place [see Fig. 3(d)]. This is an important observation because it indicates that *A. castellanii* can synthesise cellulose, in response to stressors, before fully differentiating into a cyst. Therefore, it is sensible to imply that efficacy of therapeutic intervention can be compromised even a few hours after *A. castellanii* has been exposed to chemotherapeutic treatment.

Compared to CW fluorescence (see Figs. 4(b)-(d)), the 5.8GHz distribution obtained with phonon microscopy does not appear uniformly across the cyst; instead it seems to increase (see green colour in composites from Fig. 2). This could be because the generated phonon field attenuates before propagating through the whole of the cyst or because this distribution is a consequence of the synthesis of cellulose rather than its direct measurement.

The growth of the last distribution (6.2GHz see Fig. 3(c)) corresponds well with the increased resistance to biocides (such as hydrogen peroxide and hydrochloric acid) 12-36 hours after encystation has commenced as reported by *Turner et al* [31]. There might be two possible causes for this distribution: the synthesis of the exocyst wall which is made up of an unidentified acid-resistant protein or is due to a lower water content as it shows the greatest Brillouin frequency which has been shown to be a good indicator of hydration level [30].

Increased number of samples will improve the robustness of the statistical analysis however the results presented here clearly show that the Brillouin signature of encysting *A. castellanii* changes towards higher frequencies in defined frequency distributions. We attribute these changes to dehydration, compaction and stiffening of the cysts: the Brillouin frequency depends on the refractive index and sound velocity. The latter is a function of density and elasticity which are parameters previously shown to be useful biomarkers [27–29].

## 4. Conclusion

This study presents phonon microscopy as a new imaging technique that enables fundamental research into the dynamic cytomechanical characterization of the eukaryotic organism *A. castellanii*. This approach could, ultimately, lead to the identification of novel targets for therapeutic and diagnostic applications. We have shown that synthesis of cellulose occurs soon after the encystation process has started. This is an important observation because it shows that *A. castellanii* cyst reacts quickly to adverse conditions by synthesising cellulose in the cyst wall, which increases the tolerance of the encysting organism to adverse treatment. This partially explains why *A. castellanii* cysts resistance to biocides increases over time and suggests that cellulose is the main component that enables *A. castellanii* to survive while the differentiation into a cyst is still taking place. These results show a promising potential for the application of Brillouin scattering as a contrast mechanism for biological applications and offer further opportunities for the study of fundamental biological processes in *A. castellanii* and other relevant biological organisms.
